# Antifungal susceptibility and clinical efficacy of chlorhexidine combined with topical ophthalmic medications against *Fusarium* species isolated from corneal samples

**DOI:** 10.3389/fcimb.2025.1532289

**Published:** 2025-04-15

**Authors:** Min Kang, Yuxuan Wu, Qingquan Shi, Zhiqun Wang, Yang Zhang, Kexin Chen, Xizhan Xu, Man Zhou, Qingfeng Liang, Xinxin Lu

**Affiliations:** ^1^ Department of Laboratory Medicine, Beijing Institute of Ophthalmology, Beijing Tongren Eye Center, Beijing Tongren Hospital, Capital Medical University, Beijing, China; ^2^ Beijing Institute of Ophthalmology, Beijing Tongren Eye Center, Beijing Tongren Hospital, Capital Medical University, Beijing, China

**Keywords:** fungal keratitis, *Fusarium* spp., antifungal susceptibility, chlorhexidine, clinical efficacy observation

## Abstract

**Objective:**

This study investigated the susceptibility of various *Fusarium* fungi to five topical antifungal agents: natamycin, voriconazole, chlorhexidine, natamycin combined with chlorhexidine, and voriconazole combined with chlorhexidine. And to explore the clinical feasibility of combination therapy in the treatment of corneal infections caused by *F. solani*, with the goal of optimizing the treatment regimen for fungal keratitis.

**Methods:**

A total of 194 strains of *Fusarium* were isolated from the corneas between 2013 and 2024 and identified to the species level using mass spectrometry. The MICs was determined using a commercial microdilution plate to assess the in vitro activity of the drugs used alone and in combination (natamycin/chlorhexidine, voriconazole/chlorhexidine). Additionally, the clinical efficacy was prospectively observed in 5 patients with corneal infections caused by *F. solani*. The treatment regimens included 5% natamycin combined with 0.04% chlorhexidine, chlorhexidine used alone, or natamycin used alone, with follow-up lasting up to 90 days.

**Results:**

*F. solani* species complex (FSSC, 46.91%) and the *F. fujikuroi* species complex (FFSC, 45.88%) were the predominant isolates, with a geographical distribution concentrated in Northern China. The MICs for natamycin in FSSC and FFSC were both 2–8 μg /mL, respectively. The MICs for FSSC and FFSC respectively ranged from 0.25–16 and 1–8 μg/mL for voriconazole and 2 to > 16 μg/mL and 1 to > 16 μg/mL for chlorhexidine. The MICs of natamycin were not significantly different between FSSC and FFSC. However, voriconazole, chlorhexidine, natamycin combined with chlorhexidine, and voriconazole combined with chlorhexidine had significantly higher MICs for FSSC compared with FFSC. Compared with voriconazole, voriconazole combined with chlorhexidine exhibited enhancement of antifungal activity against 100% of tested *Fusarium* strains. Compared with natamycin, enhancement of antifungal activity of natamycin combined with chlorhexidine was 81.4% for all *Fusarium* spp., and the activity were significantly lower for *F. solani* (65.9%) than for non-*F. solani* species (93.6%). Among the 5 patients, 3 patients received treatment with natamycin combined with chlorhexidine, resulting in clinical cure in 2 patients (in 1-1.5 months), while 1 patient required a corneal transplant due to delayed treatment. One patient treated with natamycin alone and one treated with chlorhexidine alone both achieved clinical cure (in 2-3 months).

**Conclusion:**

Natamycin combined with chlorhexidine and voriconazole combined with chlorhexidine exhibited enhancement of antifungal activity against *Fusarium* spp. during in vitro sensitivity tests. The findings of this study provide valuable guidance for establishing the epidemiological cutoff and clinical MIC values for *Fusarium* spp. This study paves the way for future multicenter studies on the treatment of FK with natamycin and chlorhexidine.

## 1 Introduction

Fungal keratitis (FK) is one of the leading causes of monocular blindness in developing countries where manual farming practices are predominant (Halim et al., 2021; Bisen et al., 2024). Infections caused by *Fusarium* species represent the highest proportion of FK cases (Khurana et al., 2022; Roberts et al., 2023; Skora et al., 2023). The most common route of infection is microtrauma or destructive ocular surface disease (Oliveira Dos Santos et al., 2020).

*Fusarium* species are ubiquitous filamentous fungi, commonly found in soil, plants, and water (Nucci et al., 2021). Classification of *Fusarium* spp. is complex; molecular techniques suggest that the medically relevant genus *Fusarium* consists of several species and subspecies (Sav et al., 2018). *Fusarium* spp. have shown broad resistance to different classes of antifungal drugs, and susceptibility patterns may vary within species complexes (Slavin et al., 2015). Natamycin or voriconazole has been widely used for the topical treatment of fungal keratitis (Austin et al., 2017; Edwar et al., 2020; Sharma et al., 2022).

Chlorhexidine is a cationic biguanide disinfectant and antiseptic (Hiom et al., 1996; McDonnell and Russell, 1999). Its mechanism of action involves binding to and disrupting bacterial cell membranes, with a wide range of antimicrobial activity (Lima de Sousa et al., 2024). Chlorhexidine affects the fungal plasma membrane and has significant effects elsewhere in the cell (Abbood et al., 2023). It binds to and persists with proteins in the stratum corneum of the epidermis (Sebben, 1983), which has been shown to have a cumulative effect when used repeatedly (Mullany et al., 2006).Recent studies have shown that the combination of natamycin and chlorhexidine or voriconazole and chlorhexidine can also achieve good therapeutic effects (Jiang et al., 2020; Arunga et al., 2021; Raghavan et al., 2021). However, based on published research data, there is currently a lack of systematic *in vitro* drug susceptibility tests and studies using susceptibility results to guide clinical treatment.

Thus, we designed a commercial broth microdilution plate. The plate includes five commonly used topical antifungal agents (natamycin, voriconazole, chlorhexidine, natamycin combined with chlorhexidine, and voriconazole combined with chlorhexidine). By determining the minimum inhibitory concentrations (MICs) of these agents, we aim to provide clinicians with data that can help guide therapeutic decisions and optimize treatment regimens for patients with FK. Antifungal susceptibility testing to determine the MIC ranges and antifungal activity was conducted on 194 strains of *Fusarium* isolated from corneal tissues injured during agricultural activities from 2013 to 2024. Through observations of clinical efficacy, we evaluated the feasibility of treating *Fusarium* keratitis with topical applications of natamycin combined with chlorhexidine and chlorhexidine alone.

## 2 Materials and methods

### 2.1 Strain sources

A total of 194 strains of *Fusarium* spp. were isolated from immunocompetent patients with agriculture-related injuries between 2013 and 2024. Most of the isolates were from Northern China. Identification was performed through morphological analysis, mass spectrometry. *Fusarium* strains were cultured on Potato Dextrose Agar (PDA) at 28°C for 3 days. Following incubation, slide preparations were made and stained with lactophenol cotton blue. Morphological identification (Lu et al., 2023) was performed via microscopic examination. The isolates to be identified were inoculated on PDA medium and cultured at 28°C for 3 days until colonies formed. Hyphae from the edge of the colony were selected with a pointed cotton swab, directly smeared thinly onto the target plate, and dried. 1 μL of 70% formic acid solution was added after drying, followed by 1 μL of matrix solution, which was dried again before mass spectrometry analysis. The solution was primarily a supersaturated solution of α-cyano-4-hydroxycinnamic acid, where the solvent consisted of 50% pure acetonitrile, 47.5% distilled water, and 2.5% trifluoroacetic acid. Identification was performed using the Zhuhai Dir Biotechnology Co., Ltd. fully automated microbial mass spectrometry detection system (Smart MS 5020). An identification score ≥2.0 allows identification to the species/complex level (Huang et al., 2017; Lu, 2021).

### 2.2 Classification of *Fusarium*

*Fusarium* species were classified as follows (Uemura et al., 2022):

1. *F. solani* species complex (FSSC) includes strains of *F. solani* and *F. keratoplasticum*;
2. *F. fujikuroi* species complex (FFSC) includes strains of *F. verticillioides* and *F. proliferatum*;
3. *F. oxysporum* species complex (FOSC) consists of only one species, *F. oxysporum*;
4. *F. dimerum* species complex (FDSC) consists of only one species, *F. dimerum*;
5. To more clearly determine the MICs of topical antifungal medications, *Fusarium* spp. were divided into the *F. solani* group and the non-*F. solani* group, including *F. verticillioides*, *F. proliferatum*, *F. oxysporum*, *F. keratoplasticum*, and *F. dimerum*.

### 2.3 Drug sensitivity plates

The antifungal agents included natamycin, natamycin combined with chlorhexidine, voriconazole, voriconazole combined with chlorhexidine, amphotericin B, terbinafine, posaconazole, and itraconazole. Preliminary experiments confirmed the concentration of chlorhexidine in combination with other drugs (8 μg/mL). The detection ranges for the antifungal agents were as follows: natamycin, 0.25–128 μg/mL; natamycin combined with chlorhexidine, 0.25/8–128/8 μg/mL; voriconazole, 0.015–16 μg/mL; voriconazole combined with chlorhexidine, 0.015/8–16/8 μg/mL; amphotericin B, 0.125–16 μg/mL; terbinafine and posaconazole, 0.015–16 μg/mL; and chlorhexidine and itraconazole, 0.03–16 μg/mL. The plates were customized by Zhuhai DL Biotech Co., Ltd. The reagent plates consisted of antifungal agents and a colorimetric agent. The MIC values were determined based on the color changes in the reaction wells. The colorimetric agent was Alamar Blue, which responds to changes in the redox potential caused by fungal growth in the reaction wells. The proliferation of fungi consumes molecular oxygen, causing the colorimetric agent to change color from blue to pink (the oxidized form of Alamar Blue is blue, while the reduced form is pink).

### 2.4 Confirmation of combined drug concentrations

Using a microbroth dilution method (John, 2008), 10 strains of *Fusarium* were tested, revealing a clear antifungal activity when the concentration of chlorhexidine reached 8 μg/mL in the combined antifungal susceptibility test with *F. solani*. In contrast, for non-*F. solani*, a significant antifungal activity was observed when the chlorhexidine concentration reached 1 or 2 μg/mL. Because *F. solani* accounted for over 50% of the strains studied, a chlorhexidine concentration of 8 μg/mL was selected for combination with natamycin and voriconazole. In the combined drug susceptibility tests, when the concentration of chlorhexidine reached 8 μg/mL, the MICs of natamycin and voriconazole in combination with chlorhexidine were lower than the MICs of the corresponding individual drugs, indicating enhancement of antifungal activity.

### 2.5 Antifungal susceptibility testing and interpretation criteria

Strains were inoculated onto PDA and incubated at 28°C for 5 d. A suitable amount of spores was scraped and resuspended in 5 mL of diluent (containing 0.1% Tween and 0.85% sterile saline). The cell concentration was adjusted to 1–5×10^8^ cfu/mL. A 100-μL aliquot of this suspension was added to 10 mL of RPMI 1640 solution supplemented with glucose, achieving a final *Fusarium* spore concentration of 1–5×10^4^ cfu/mL. To each well of a 96-well plate, 100 μL of the suspension was added. After incubating the plates at 35°C for 48 h, the MIC was determined by identifying the lowest concentration of antifungal agent required to achieve 100% inhibition of fungal growth compared to the control (indicated by a purple or red color). The criteria for synergy were based on a custom susceptibility plate; synergy was confirmed when the MIC of the combination of natamycin or voriconazole with 8 μg/mL chlorhexidine was lower than that of natamycin or voriconazole alone. We also included the *Aspergillus flavus* strain ATCC 204304 for quality control in the analysis. The methods for the antifungal susceptibility tests were based on CLSI M38-A2 (John, 2008).

### 2.6 Evaluation of clinical efficacy

Patients were enrolled in September 2023 during their first visit to our center. The inclusion criterium was a positive culture for *Fusarium* spp. The diagnostic criteria included the presence of hyphal structures in corneal scrapings, fungal hyphae images visible during *in vivo* confocal microscopy (IVCM), *Fusarium* spp. found in the culture of the corneal specimen, and susceptibility testing performed. Treatment involved a combination of 5% natamycin and 0.04% chlorhexidine eye drops along with 0.04% chlorhexidine eye drops alone. Follow-up assessments were conducted at 7, 14, 21, 30, 60, and 90 d. The observation endpoints were clinical cure (Rahman et al., 1998), defined by corneal epithelial healing, complete control of infection, no recurrence within 2 months of follow-up, or the need for corneal transplantation. Four patients with *Fusarium* keratitis were enrolled.

### 2.7 Statistical methods

Statistical analyses were conducted using R version 4.4.1 (R Foundation, Vienna, Austria). Categorical variables were expressed as frequency and percentage. The MIC_50_/MIC_90_ and frequency of studied isolates were determined (Soleimani et al., 2023). Mann–Whitney U test was used to compare resistance by region and the MIC values between the *F. solani* and non-*F. solani* groups. Kruskal–Wallis’s test and Dunn’s test were used to analyze MIC values among different complexes. *P* < 0.05 was considered statistically significant.

## 3 Results

### 3.1 Classification, geographic distribution, and detection time of *Fusarium* spp.

**Figure 1:**
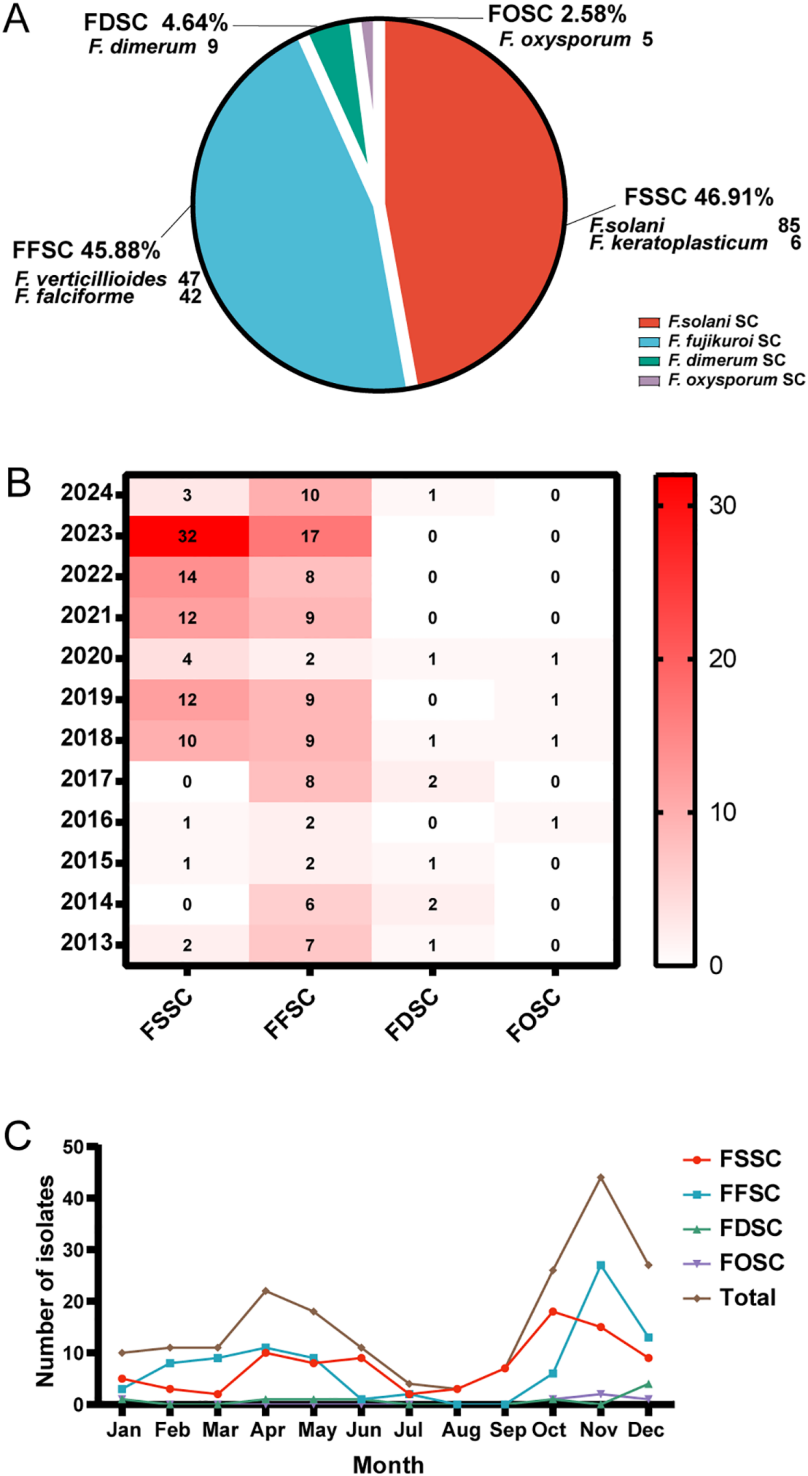
Classification of *Fusarium* isolates and their annual and seasonal distributions. **(A)** Distribution of 194 *Fusarium* isolates. SC = species complex. **(B)** Annual distribution heat map of *Fusarium* spp. complexes from 2013 to 2024. The number of *Fusarium* species complexes are shown per year within the squares of the heat map. The color scale indicates number of isolates and the darker the red color, the higher the number of isolates while white represents 0 isolates. **(C)** Seasonal distributions of *Fusarium* spp. complexes. Total represents the number of *Fusarium* isolates.

A total of 194 *Fusarium* strains were isolated from 194 agricultural immunocompetent workers between 2013 and 2024, all of whom had injuries from crops. The most common isolate was *F. solani* (*n* = 85, 43.81%) followed by *F. verticillioides* (*n* = 47, 24.23%), *F. proliferatum* (*n* = 42, 21.65%), *F. keratoplasticum* (*n* = 6, 3.09%), *F. dimerum* (*n* = 9, 4.64%), and *F. oxysporum* (*n* = 5, 2.58%). The isolates were categorized into their respective species complexes (**Figure 1A**), with FSSC being the most common (*n* = 91, 46.91%) followed by FFSC (*n* = 89, 45.88%), FDSC (*n* = 9, 4.64%), and FOSC (*n* = 5, 2.58%). Geographically, *Fusarium* spp. was concentrated in Beijing, Hebei, Inner Mongolia, and Liaoning, almost located in Northern China (**Supplementary Appendix Figure 1**). No significant temporal or seasonal differences in frequency were observed among the *Fusarium* species complexes (**Figures 1B, C**). The incidence of *Fusarium* spp. peaked from March to June and from September to December, with significant increases observed in autumn and spring (**Figure 1C**).

### 3.2 Comparison of MICs of antifungal drugs for *Fusarium* species complexes

**Figure 2:**
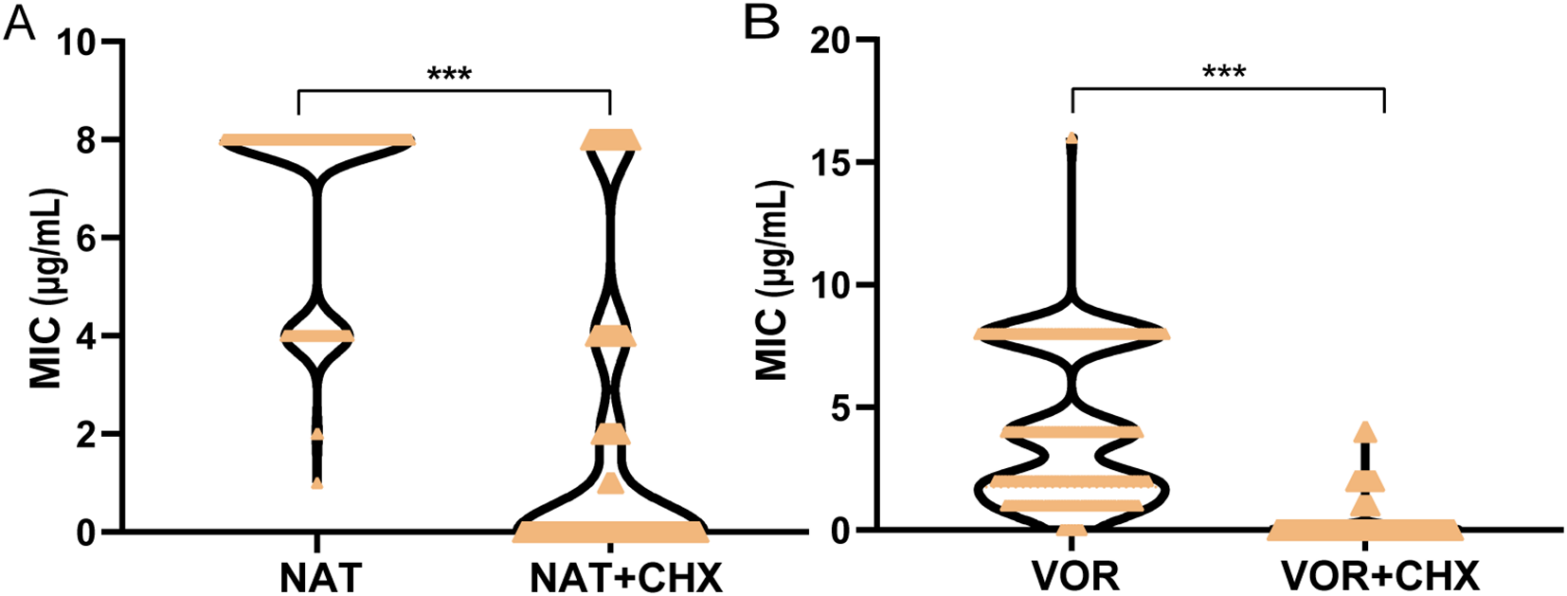
Comparison of the MIC values of four antifungal drugs for *Fusarium* species. Violin plots illustrating the distributions of MICs for **(A)** natamycin (NAT) vs. natamycin combined with chlorhexidine (NAT+CHX), **(B)** voriconazole (VOR) vs. voriconazole combined with chlorhexidine (VOR+CHX) against *Fusarium* species. Asterisks denote statistical significance levels: ***P < 0.001.

*In vitro* susceptibility tests for *Fusarium* isolates showed that the MIC values of natamycin combined with chlorhexidine (<0.12/8-8/8μg/mL) was significantly lower than that of natamycin (8-8μg/mL) and the MICs of voriconazole combined with chlorhexidine (<0.015-4μg/mL)was lower than that of voriconazole (0.25-8μg/mL) (*P*<0.001, *P*<0.001) (**Figures 2A, B**; **Supplementary Appendix Table 1**).

#### 3.2.1 Natamycin and natamycin combined with chlorhexidine

**Figure 3:**
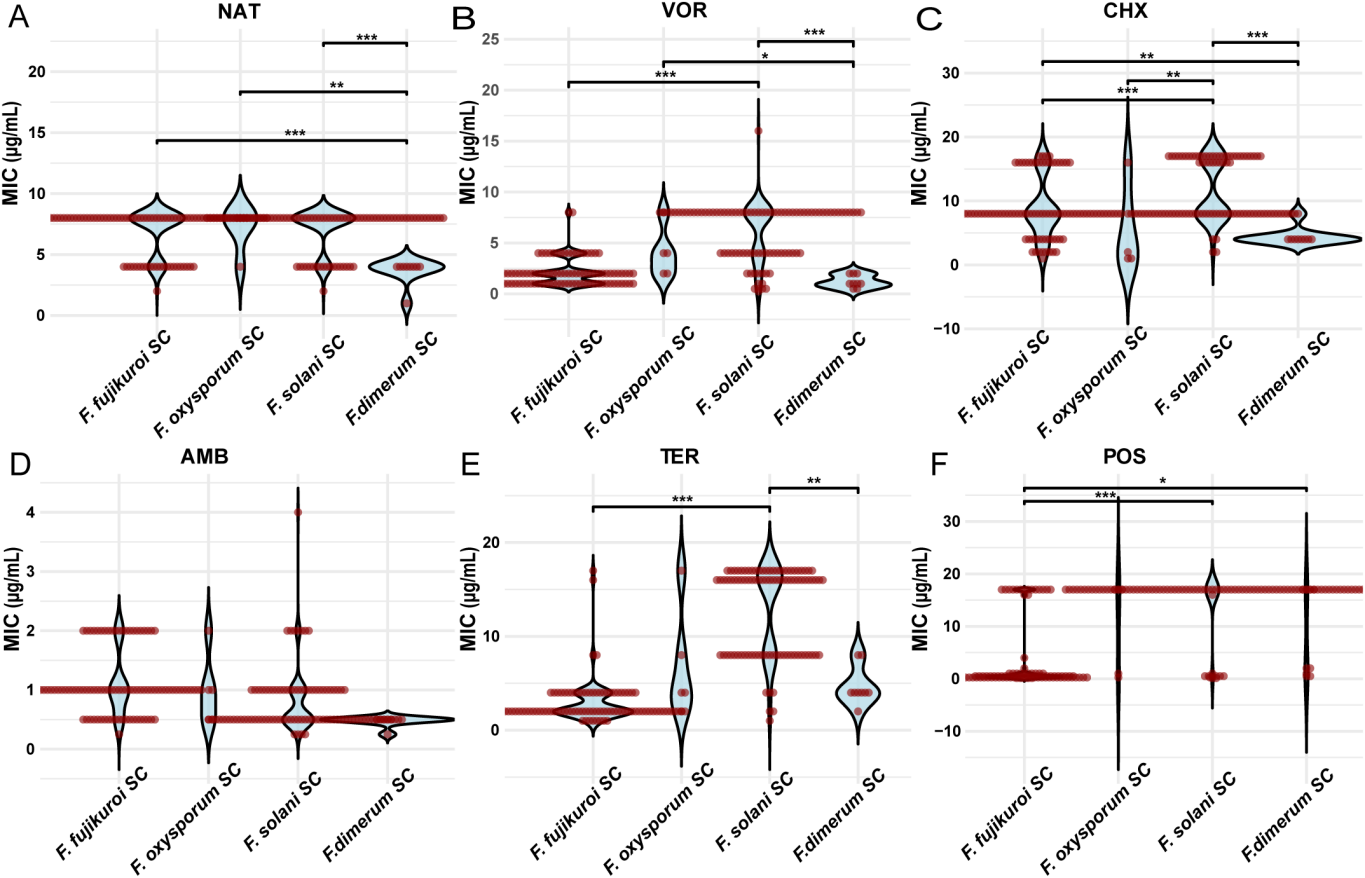
Comparison of the MIC values of six antifungal drugs for different *Fusarium* species complexes. MIC distributions of **(A)** natamycin (NAT), **(B)** voriconazole (VOR), **(C)** chlorhexidine (CHX), **(D)** amphotericin B (AMB), **(E)** terbinafine (TER), and **(F)** posaconazole (POS) across FSSC, FFSC, FDSC and FOSC. Asterisks denote statistical significance levels: *P < 0.05, **P < 0.01, and ***P < 0.001.

The MICs of natamycin were lower for FDSC compared with FSSC, FFSC, and FOSC (*P* < 0.001, *P* < 0.001, and *P* < 0.01, respectively). Natamycin combined with chlorhexidine showed significantly higher MIC values in FSSC than in FFSC (*P* < 0.01) (**Table 1**; **Figure 3A**).

**Table 1:** MIC_50_, MIC_90_, and MIC ranges of different *Fusarium* spp. complexes.

	FSSC (μg/mL)			FFSC (μg/mL)			FDSC (μg/mL)			FOSC (μg/mL)		
	MIC_50_	MIC_90_	Range	MIC_50_	MIC_90_	Range	MIC_50_	MIC_90_	Range	MIC_50_	MIC_90_	Range
NAT	8	8	2-8	4	8	2-8	4	4	1-4	8	8	4-8
NAT/CHX	1/8	8/8	<0.12/8-8/8	<0.12/8	4/8	<0.12/8-8/8	<0.12/8	<0.12/8	<0.12/8	<0.12/8	0.25/8	<0.12/8-0.25/8
VOR	8	8	0.25-16	2	4	1-8	1	2	0.5-2	4	8	2-8
VOR/CHX	0.03/8	2/8	<0.015/8-4/8	<0.015/8	0.25/8	<0.015/8-2/8	<0.015/8	<0.015/8	<0.015/8	<0.015/8	0.5/8	<0.015/8-0.5/8
CHX	8	>16	2->16	8	16	1->16	4	4	4-8	2	16	1-16
AMB	0.5	1	0.25-4	1	2	0.25-2	0.5	0.5	0.25-0.5	1	2	0.5-2
TER	16	>16	1->16	2	4	1->16	4	4	2-8	4	4	2->16
POS	>16	>16	0.125->16	0.5	>16	0.125->16	2	>16	0.5->16	>16	>16	0.25->16
ITR	>16	>16	0.125->16	>16	>16	0.5->16	>16	>16	>16	>16	>16	>16

NAT, natamycin; NAT/CHX, natamycin/chlorhexidine; VOR, voriconazole; VOR/CHX, voriconazole/chlorhexidine; CHX, chlorhexidine; AMB, amphotericin B; TER, terbinafine; POS, posaconazole; ITR, itraconazole.

#### 3.2.2 Voriconazole and voriconazole combined with chlorhexidine

Voriconazole had significantly higher MIC values in FSSC compared to FFSC and FDSC (*P* < 0.001 and *P* < 0.001, respectively). Voriconazole combined with chlorhexidine also showed significantly higher MIC values in FSSC compared to FFSC (*P* < 0.001; **Table 1**; **Figure 3B**).

#### 3.2.3 Chlorhexidine

The MIC values of chlorhexidine were significantly higher in FSSC than in FFSC. Specifically, MIC exceeded 16 μg/mL for 28 strains in FSSC but only four strains in FFSC (*P* < 0.001; **Table 1**; **Figure 3C**).

#### 3.2.4 Other antifungal drugs

Amphotericin B had significantly higher MIC values in FFSC compared with FSSC (*P* < 0.001) and FDSC (*P* < 0.001). Terbinafine showed significantly higher MIC values in FSSC compared with FFSC (*P* < 0.001) and FDSC (*P* < 0.01). Posaconazole had significantly higher MIC values in FSSC compared with FFSC (P < 0.001). For itraconazole, 97.9% of *Fusarium* isolates had MIC values exceeding 16 μg/mL (**Table 1**; **Figures 3D-F**).

### 3.3 Differences in the MIC values of topical ophthalmic medications between *F. solani* and non-*F. solani*

**Figure 4:**
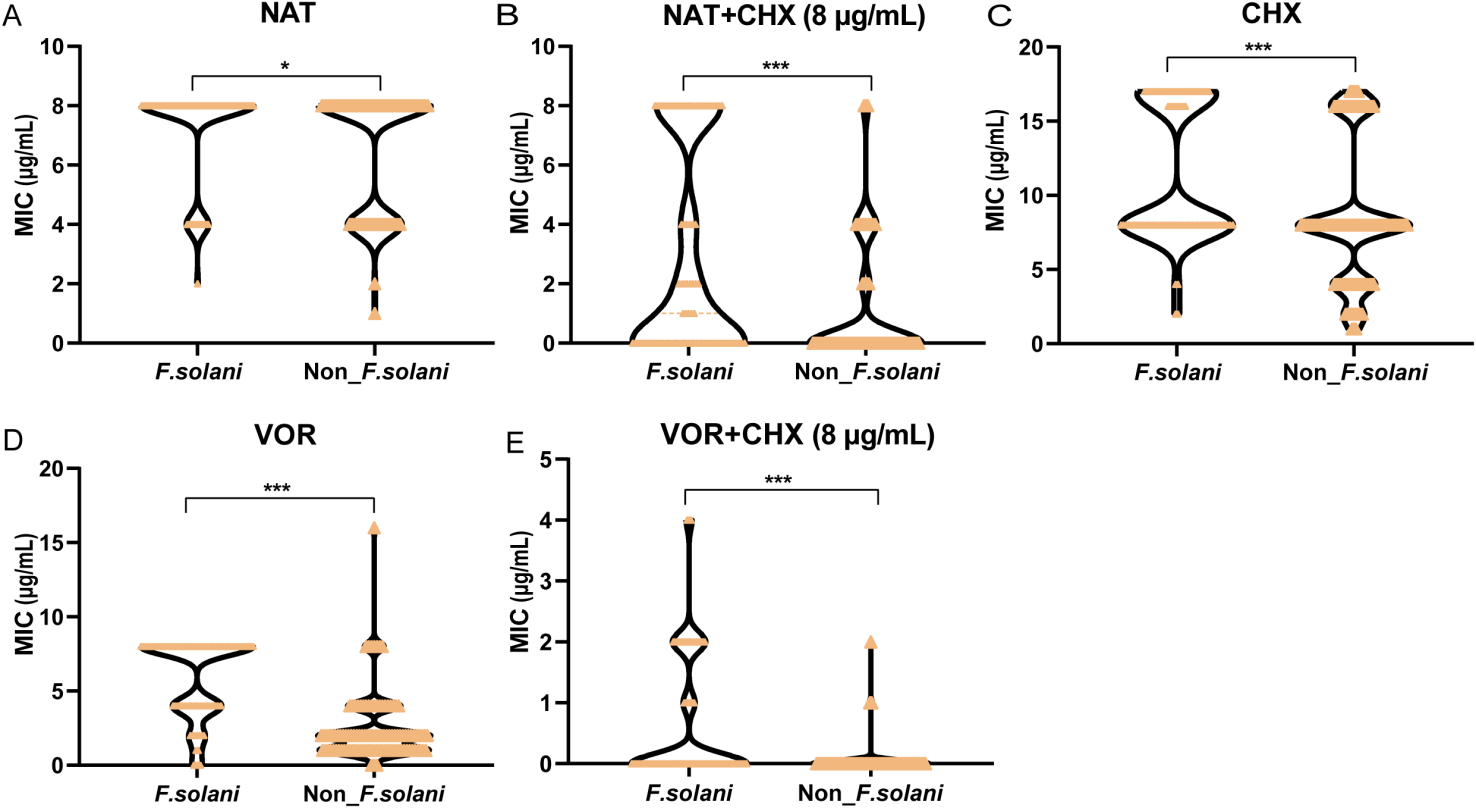
Comparison of topical medication efficacy between *F. solani* and non-*F. solani* species. Violin plots illustrating the distributions of MICs for **(A)** natamycin (NAT), **(B)** natamycin combined with chlorhexidine (NAT+ CHX), **(C)** chlorhexidine (CHX), **(D)** voriconazole (VOR), and **(E)** voriconazole combined with chlorhexidine (VOR+ CHX) against *F.solani* and non-*F. solani* species. CHX concentration in combination drug was fixed at 8 µg/mL. Asterisks denote statistical significance levels: *P < 0.05, and ***P < 0.001.

The MIC values for *F.solani* and non-*F. solani* respectively ranged from 2–8 and 1–8 μg/mL for natamycin; 0.5–8 and 0.25–16 μg/mL for voriconazole; 2 to > 16 and 1 to > 16 μg/mL for chlorhexidine; < 0.12/8 to 8/8 and < 0.12/8 to 8/8 μg/mL for natamycin combined with chlorhexidine; and < 0.015/8 to 4/8 and < 0.015/8 to 2/8 μg/mL for voriconazole combined with chlorhexidine (**Table 2**). The MIC values of natamycin, voriconazole, chlorhexidine, natamycin combined with chlorhexidine (8μg/mL), and voriconazole combined with chlorhexidine (8μg/mL) were significantly higher in the *F. solani* group than in the non-*F. solani* group (*P* < 0.05, *P* < 0.001, *P* < 0.001, *P* < 0.001, and *P* < 0.001, respectively; **Figures 4A–E**). Compared with natamycin, natamycin combined with chlorhexidine exhibited enhancement of antifungal activity against 81.4% of 194 *Fusarium* strains. For *F. solani*, the enhancement of antifungal activity was 65.9% (56/85), significantly lower than that for non-*F. solani* (93.6%, 102/109; *P* < 0.01). For *F. solani*, 45.9% (39/85) of clinical isolates showed the lowest concentration in natamycin combined with chlorhexidine (8μg/mL), significantly lower than that for non-*F. solani* (68.8%, 75/109; P < 0.01; **Supplementary Appendix Table 3**). In contrast, compared with voriconazole, voriconazole combined with chlorhexidine (8μg/mL) exhibited a 100% enhancement of antifungal activity. For *F. solani*, 48.2% (41/85) of clinical isolates showed the lowest concentration, significantly lower than that for non-*F. solani* (76.1%, 83/109; P < 0.001; **Supplementary Appendix Table 3**).

**Table 2:** MIC_50_, MIC_90_, and MIC ranges of ophthalmic antifungal drugs against *F. solani* and non*-F.solani* species.

	*F.solani* (μg/mL)			Non*-F.solani* (μg/mL)			P value
	MIC_50_	MIC_90_	Range	MIC_50_	MIC_90_	Range	
NAT	8	8	2-8	8	8	1-8	0.05
NAT/CHX	1/8	8/8	<0.12/8-8/8	<0.12/8	4/8	<0.12/8-8/8	0.001
VOR	8	8	0.5-8	2	4	0.25-16	0.001
VOR/CHX	0.03/8	2/8	<0.015/8-4/8	<0.015/8	0.25/8	<0.015/8-2/8	0.001
CHX	8	>16	2->16	8	16	1->16	0.001

NAT, natamycin; NAT/CHX, natamycin/chlorhexidine; VOR, voriconazole; VOR/CHX, voriconazole/chlorhexidine; CHX, chlorhexidine.

### 3.4 Clinical efficacy observation

In Northern China, topical voriconazole formulations are limitedly available, and natamycin ophthalmic solution is often the first-line of treatment chosen by clinicians for *Fusarium* keratitis. This study was a single-center study, and only 5 patients diagnosed with *F. solani* keratitis were collected during the study period without other antifungal treatments. Clinical data from 5 patients is a limitation of this study since statistically significant conclusions can’t be established. Among five patients, three patients (Case 1-Case 3) received topical treatment with 5% natamycin combined with 0.04% chlorhexidine, and one patient (Case 4) received only natamycin, while one patient (Case 5) received only chlorhexidine. The results are detailed below.

#### 3.4.1 Case 1

**Figure 5:**
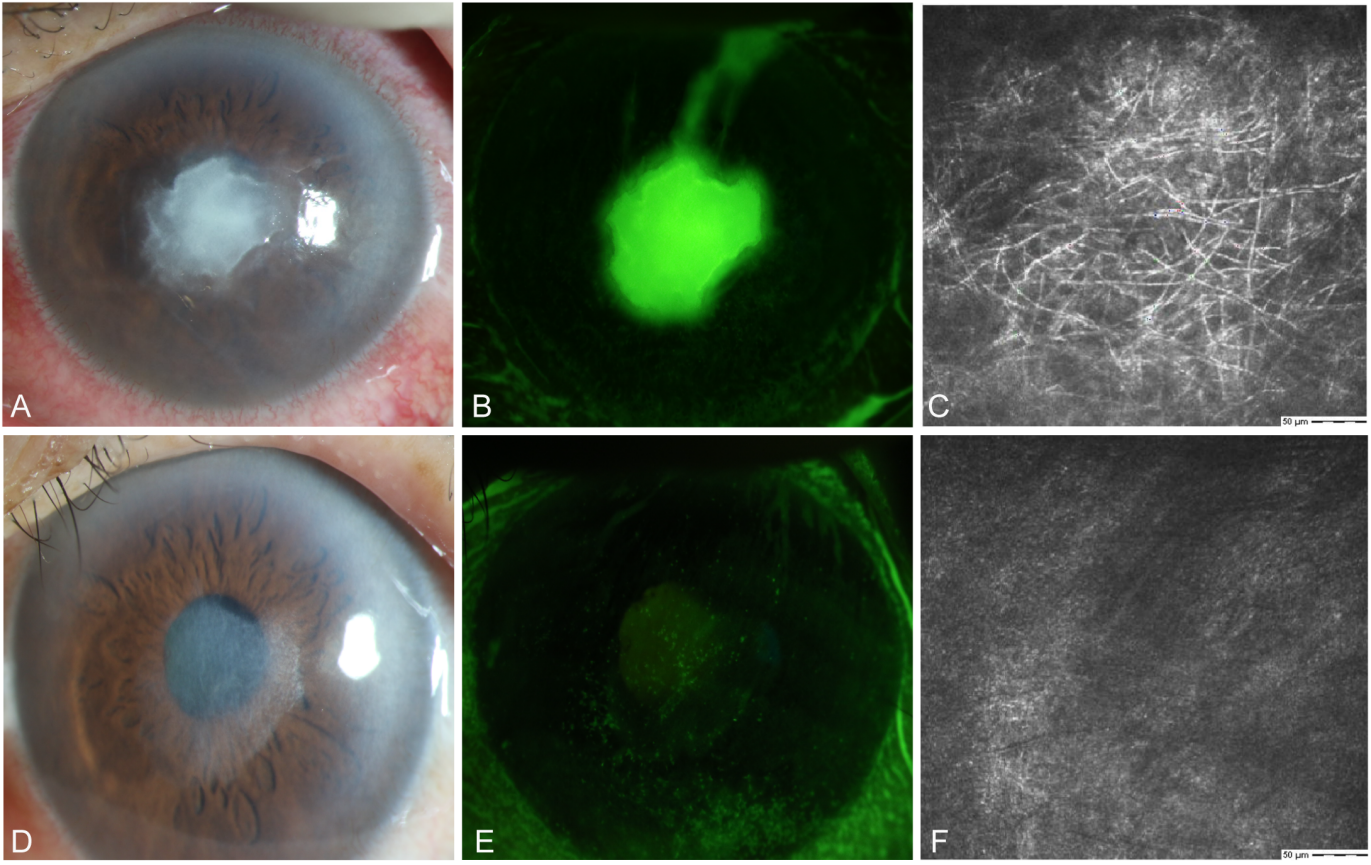
Slit-lamp and IVCM images acquired during patient (Case 1) treatment. **(A)** Slit-lamp examination images obtained at initial diagnosis showing grayish-white dense infiltrates and feathery margins. **(B)** Corneal fluorescein staining images showing epithelial ulcers. **(C)** IVCM images obtained at initial diagnosis showing fungus-like structures. **(D, E)** Slit-lamp examination and corneal fluorescein staining images obtained after treatment showing corneal ulcer healing. **(F)** IVCM examination images showing corneal epithelial healing and absence of mycelial structures.

A 64-year-old female farmer presented to our hospital in September with eye pain and redness after being scratched by a bean sprout in her left eye. Slit lamp examination revealed conjunctival and ciliary congestion without scleral congestion, corneal ulcer, infiltration, stromal edema, and wrinkling of the posterior elastic layer with poorly defined borders. IVCM examination showed epithelial loss with numerous fungal hyphal structures in the ulcer area. The edges of the ulcer demonstrated significant inflammatory cell infiltration. The patient was clinically diagnosed with *Fusarium* keratitis in the left eye (**Figures 5A–C**). Treatment with 5% natamycin combined with 0.04% chlorhexidine eye drops was administered every hour for 1 month. The conjunctival congestion and edema subsided, the corneal lesions disappeared, and complete epithelial healing was achieved, leaving only superficial opacification. IVCM examination showed complete healing of the corneal epithelial ulcer, fibroblast proliferation in the stroma, and no remaining fungal hyphal structures (**Figures 5D–F**). Thus, the patient achieved clinical cure.

#### 3.4.2 Case 2

**Figure 6:**
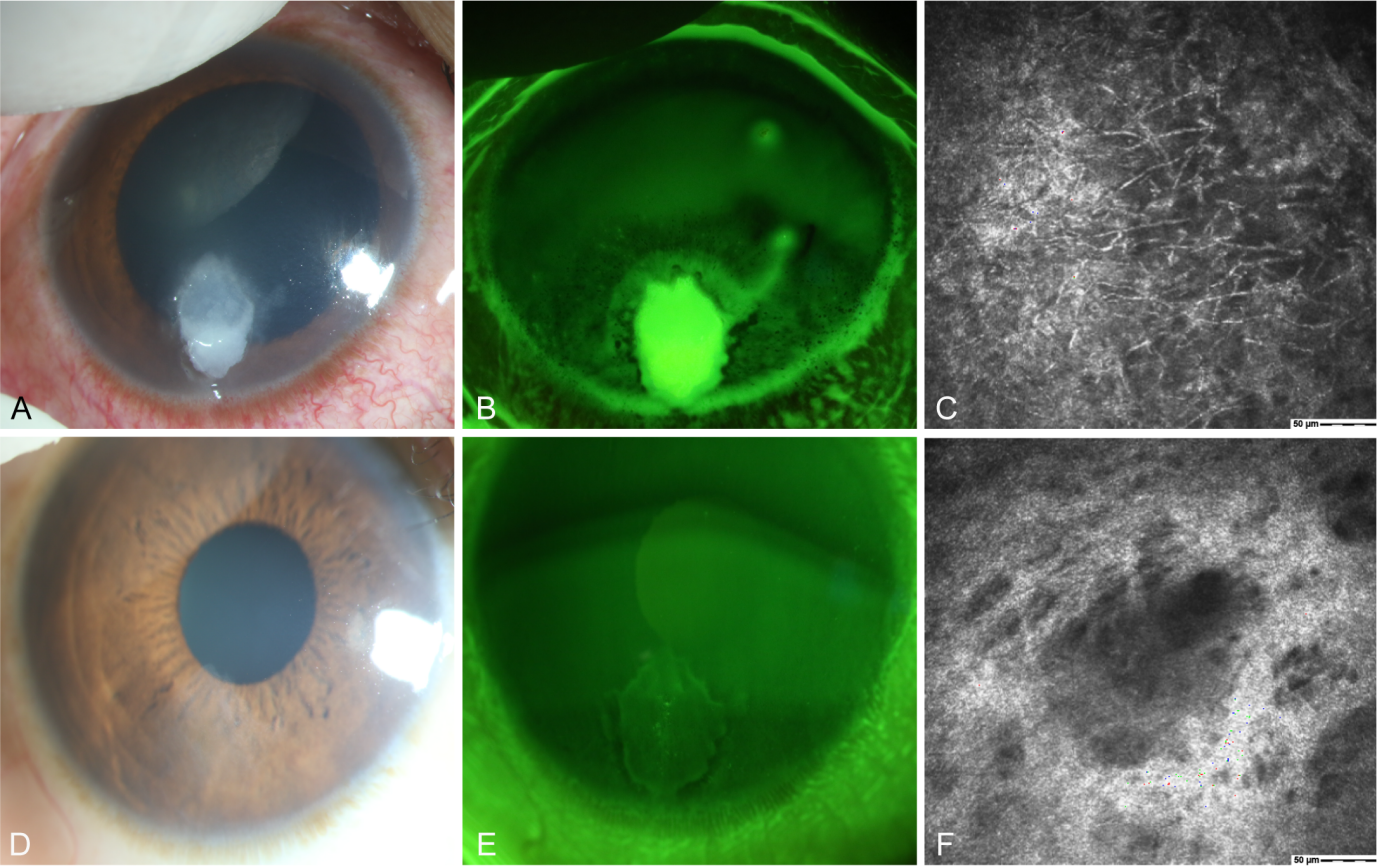
Slit-lamp and IVCM images acquired during patient (Case 2) treatment. **(A)** Slit-lamp examination images obtained at initial diagnosis showing grayish-white dense infiltrates and feathery margins. **(B)** Corneal fluorescein staining images showing epithelial ulcers. **(C)** IVCM images obtained at initial diagnosis showing fungus-like structures. **(D, E)** Slit-lamp examination and corneal fluorescein staining images obtained after treatment showing corneal ulcer healing. **(F)** IVCM examination images showing corneal epithelial healing and absence of mycelial structures.

A 50-year-old female presented with the sensation of a foreign body in her right eye after riding an electric bicycle. The patient was clinically diagnosed with right eye *Fusarium* keratitis (**Figures 6A–C**). Treatment with 5% natamycin combined with 0.04% chlorhexidine eye drops was administered every hour for 1.5 months. The conjunctival congestion and edema subsided, and complete epithelial healing was achieved, leaving only superficial opacification. IVCM examination showed complete healing of the corneal epithelial ulcer, fibroblast proliferation in the stroma, and no remaining fungal hyphal structures (**Figures 6D–F**). Thus, the patient achieved clinical cure.

#### 3.4.3 Case 3

**Figure 7:**
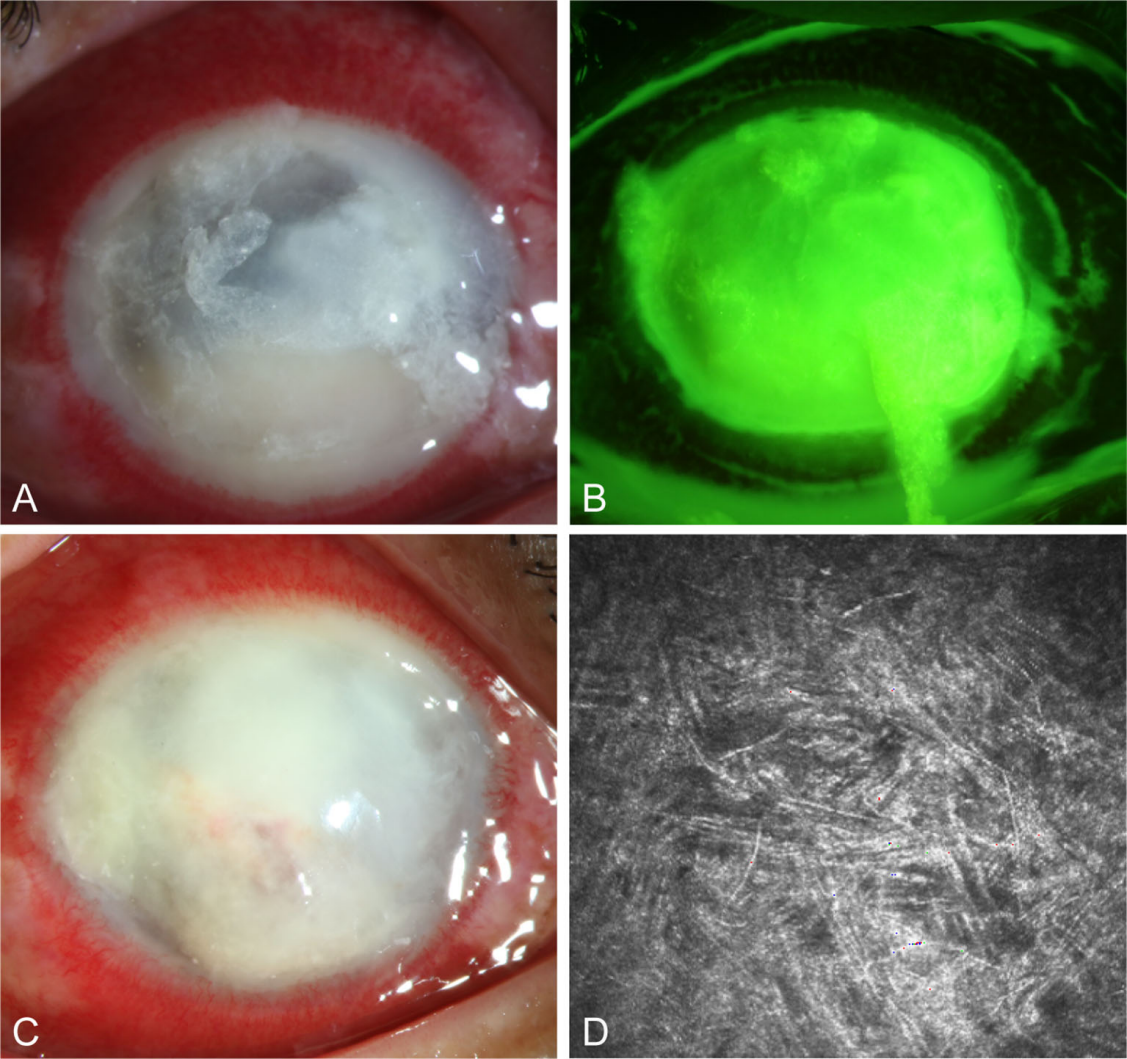
Slit-lamp and IVCM images acquired during patient (Case 3) treatment. **(A, B)** Slit-lamp examination and corneal fluorescein staining images obtained at initial diagnosis showing total corneal ulcer. **(C)** Slit-lamp examination image obtained after therapy showing total corneal ulcer. **(D)** IVCM image obtained initial diagnosis showing fungus-like structures.

A 57-year-old male with an unknown occupation presented with a total corneal ulcer. IVCM examination revealed fungal hyphae within approximately 400 microns of the stromal layer (**Figures 7A, B, D**). Following 2 weeks of treatment with 5% natamycin in combination with 0.04% chlorhexidine eye drops every hour (**Figure 7C**), the disease progressed in an uncontrolled manner, and a subsequent corneal transplantation was carried out.

#### 3.4.4 Case 4

**Figure 8:**
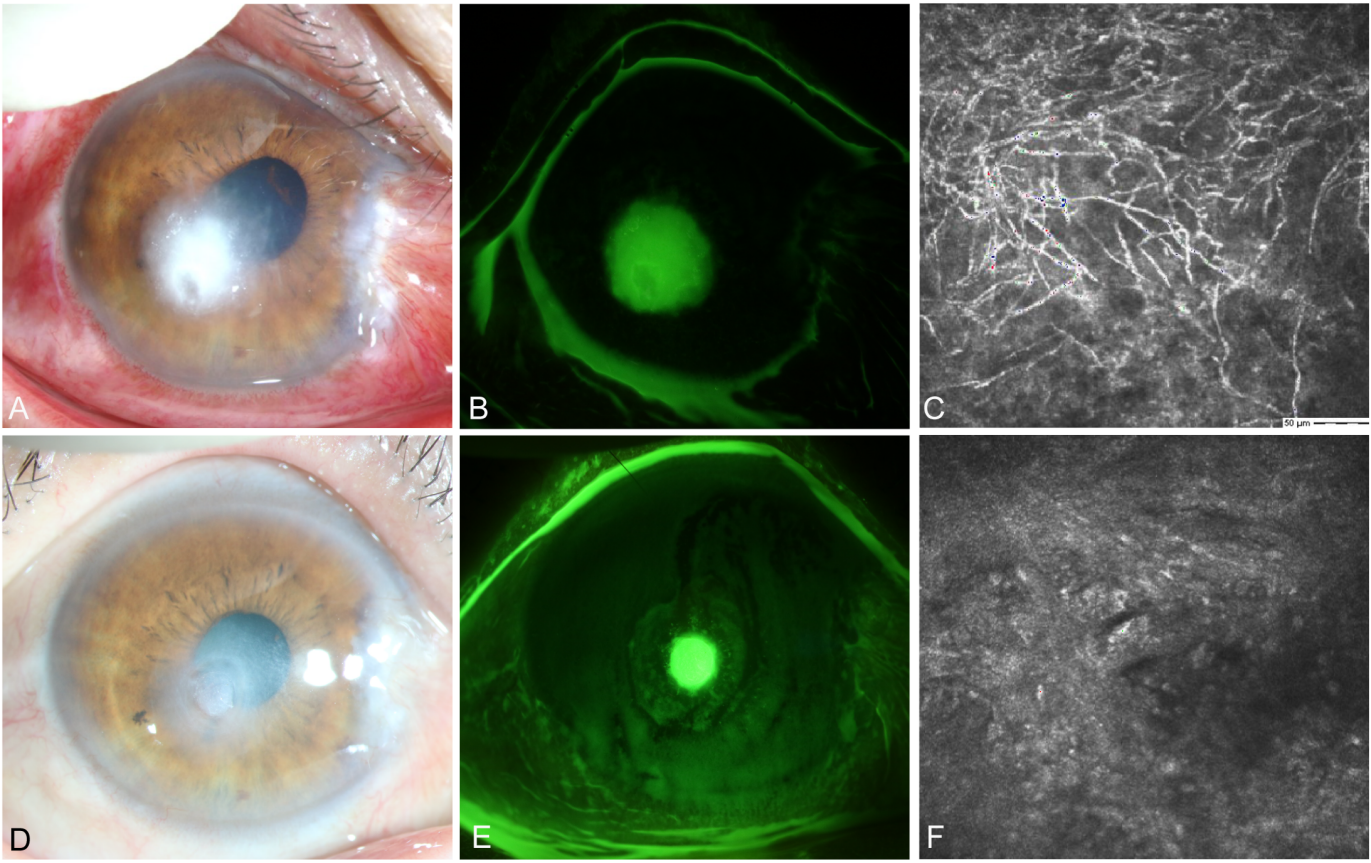
Slit-lamp and IVCM images acquired during patient (Case 4) treatment. **(A)** Slit-lamp examination images obtained at initial diagnosis showing grayish-white dense infiltrates and feathery margins. **(B)** Corneal fluorescein staining images showing epithelial ulcers. **(C)** IVCM images obtained at initial diagnosis showing fungus-like structures. **(D, E)** Slit-lamp examination and corneal fluorescein staining images obtained after treatment showing corneal ulcer healing. **(F)** IVCM examination images showing corneal epithelial healing and absence of mycelial structures.

A female farmer presented in October with redness and pain in her right eye after being scratched by corn leaves. Slit lamp examination revealed conjunctival and ciliary congestion without scleral congestion, corneal ulceration, turbidity, stromal edema, and wrinkling of the posterior elastic layer with poorly defined borders. IVCM examination showed epithelial loss with numerous fungal hyphal structures in the ulcer area along with significant inflammatory cell infiltration at the ulcer margins. The patient was clinically diagnosed with *Fusarium* keratitis in the right eye (**Figures 8A–C**). Treatment with 0.04% chlorhexidine eye drops was administered every hour for 3 months. Follow-up IVCM examination showed gradual healing of the epithelial defect, fibroblast proliferation in the stroma, and no remaining fungal hyphal structures. Thus, the patient achieved clinical cure.

#### 3.4.5 Case 5

**Figure 9:**
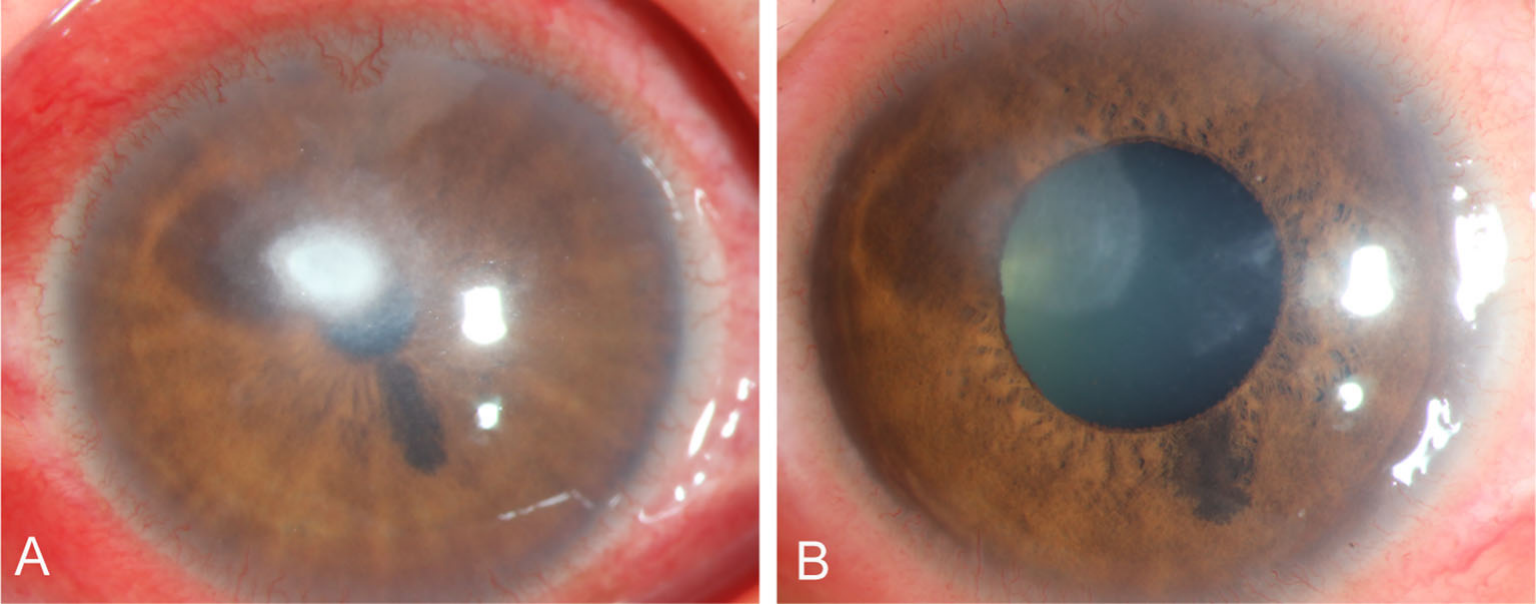
Slit-lamp images acquired during patient (Case 5 ) treatment. **(A)** Slit-lamp examination images obtained at initial diagnosis showing total corneal ulcer. **(B)** Slit-lamp examination obtained after treatment showing corneal ulcer healing.

A female farmer presented with redness and pain in her right eye, with no history of corneal trauma or foreign body involvement. Slit lamp examination revealed conjunctival congestion and edema, corneal ulceration and infiltration (**Figure 9A**) leading to the clinical diagnosis of right eye *Fusarium* keratitis. Treatment was implemented using 5% natamycin eye drops every hour for a duration of 2 months. Follow-up slit-lamp examination revealed that conjunctival congestion and edema subsided and corneal lesions disappeared (**Figure 9B**). The patient achieved clinical cure.

In this study, among 3 patients (Cases 1 - 3) receiving treatment of natamycin combined with chlorhexidine, two achieved clinical cure within 1 - 1.5 months. One patient’s drug treatment failed due to a delayed diagnosis. Additionally, there was one case where a patient treated with only natamycin was cured within two months, and yet another patient, who was treated solely with chlorhexidine, achieved clinical cure within three months.

## 4 Discussion

The specific *Fusarium* strains isolated from FK specimens depend on factors such as the geographic region, environment, and climate. Most cases in this study were patients with normal immune function from the northern regions of China, where the peak incidence of *Fusarium* spp. occurs in autumn. Among the isolates, *F. solani* was the most common (43.8%) followed by *F. verticillioides* (24.2%) and *F. proliferatum* (21.6%). In contrast, a 2008 study conducted in Shandong Province, China identified *F. solani* as the most common species followed by *F. verticillioides* and *F. oxysporum* (Xie et al., 2008), while *F. falciforme* and *F. keratoplasticum* were identified as the primary species in a 2022 study conducted in Taiwan (Huang et al., 2022). A 2010 study conducted in the United States indicated that contact lens-related keratitis was primarily caused by *F. solani* and *F. oxysporum* (Sun et al., 2010). In a study conducted in the Netherlands from 2005–2016, *F. oxysporum* was predominant followed by *F. solani* (Oliveira Dos Santos et al., 2020). A 2024 multicenter study conducted in Romania found that *F. solani* was the most common filamentous fungus in infectious keratitis, particularly during busy farming seasons (Balasoiu et al., 2024). Despite these differences in the predominant *Fusarium* species in keratitis across different regions, *F. solani* remains the primary species associated with agricultural injuries in most areas. Thus, for clinical decision-making, it is crucial to inquire about injury history and promptly identify the *Fusarium* spp. involved.

Natamycin is the most commonly used topical antifungal agent for FK in clinical practice; natamycin is the only drug currently approved by the United States Food and Drug Administration for the treatment of FK (Dennyson Savariraj et al., 2021; Arboleda and Ta, 2024). In the present study, the MIC values of natamycin ranged from 1–8 μg/mL, consistent with the range of 0.5–16 μg/mL reported by Tuft et al. (2024). In this study, the MIC was higher in the *F. solani* group than in the non-*F. solani* group, indicating worse prognosis for *F. solani* FK compared with non-*F. solani* FK. For the species complexes, the MIC of FDSC was significantly lower than those of the other complexes.

The MIC values of voriconazole differed from those of natamycin and showed greater variability among the species complexes. The MIC_50_ value of FSSC for voriconazole was higher than that of non-FSSC (8 vs. 2 μg/mL). Similarly, the MIC of voriconazole in the *F. solani* group was higher than that in the non-*F. solani* group (8 vs. 2 μg/mL). Similarly, other studies have found a relatively high MIC of voriconazole for FSSC (Walther et al., 2017; Oliveira Dos Santos et al., 2020), which was associated with delayed corneal epithelial healing and a higher incidence of complications compared with other species complexes (Oechsler et al., 2013).

Chlorhexidine is a common, inexpensive, safe, and effective antimicrobial agent with good antibacterial properties (Ward et al., 2023). Chlorhexidine binds to cell membranes to prevent pathogen adhesion and promote the release of their contents (Letzelter et al., 2019). In this study, the MIC values of chlorhexidine were significantly higher for FSSC compared to FFSC, with the MIC of chlorhexidine for *F. solani* being higher in the *F. solani* group than in the non*-F. solani* group, consistent with the findings of Oliveira Dos Santos et al. (2019). Considering the differences in the sensitivity of different *Fusarium* spp. to chlorhexidine, in the clinical treatment of *Fusarium* keratitis, detailed species identification should be prioritized before using chlorhexidine to optimize the treatment plan and improve the success rate. Such species identification is crucial for reducing treatment delays and improving prognosis.

*In vitro* testing showed significant enhancement of antifungal activity for combinations of natamycin with chlorhexidine and voriconazole with chlorhexidine. The enhancement of antifungal activity of natamycin combined with chlorhexidine was 81.4% in *Fusarium* species but only 28.9% in *F. solani*, significantly lower than that in the non-*F. solani* group (52.6%). This further demonstrates that accurate species identification is essential when using natamycin for treatment. The combination of voriconazole with chlorhexidine showed a 100% enhancement of antifungal activity. Based on 20 strains of *Fusarium* spp. collected from the skin, ear canals, and corneas of patients in Zhejiang, China, Jiang et al. (2020) found that the synergistic activity of natamycin combined with chlorhexidine was 10%, different from our findings. This suggests that factors such as the infection site, geographic location, and prior treatment can lead to variability in drug sensitivity. All strains in this study were isolated from patients with untreated agricultural injuries. Chlorhexidine is an inexpensive and easily accessible drug for the treatment of patients with FK. When combined with topical antifungal drugs, chlorhexidine can shorten the treatment time, facilitate patient management, and reduce the economic burden on patients.

Treatment with 5% natamycin combined with 0.04% chlorhexidine demonstrated good efficacy in cases 1 and 2 since the corneal lesions healed rapidly.Therefore, the combination of natamycin and chlorhexidine showed good therapeutic potential against *Fusarium* infections. The desired effect is observed in the limited data, which can provide a basis for subsequent research. The patient in case 3 presented with full corneal ulceration, after the opportunity for early drug treatment had passed. This led to uncontrolled progression of the disease, and the patient ultimately required a corneal transplant. This case highlights the importance of early diagnosis and treatment. Currently, there is no voriconazole eye drop formulation available in Northern China; if natamycin proves ineffective, voriconazole combined with chlorhexidine could be considered. In case 4, clinical cure was achieved solely with chlorhexidine; thus, in the absence of antifungal agents, chlorhexidine eye drops can be used to treat FK. Hoffman et al. (2022) showed that participants in the natamycin group had significantly better visual acuity at 90 days than the chlorhexidine group, and that natamycin was associated with faster epithelial reformation and slightly smaller scar or infiltrate size from day 7 onwards. Arunga et al. (2021) have mentioned that chlorhexidine 0.2% was found to be a useful adjunctive topical antifungal in cases of fungal keratitis not responding to natamycin 5%. This suggests that the combination of chlorhexidine and natamycin may help enhance natamycin antifungal susceptibility to *Fusarium*, potentially shortening the duration of treatment. This study will continue to accumulate data and further conduct multicenter research on the combination of natamycin and chlorhexidine.

Our study still had several limitations. First, the sample size for clinical efficacy observation was limited. This is because our laboratory is a regional tertiary hospital, and it is relatively difficult to access patients who have not previously received drug treatment. Although we obtained the MIC values of natamycin and the combination of natamycin and chlorhexidine, the clinical efficacy observation only included 5 cases, which may not be representative of the overall patient population. Despite these limitations, larger cohorts can help to confirm our findings and provide stronger evidence for the treatment of *Fusarium* keratitis.

## 5 Conclusion

Natamycin combined with chlorhexidine and voriconazole combined with chlorhexidine exhibited enhancement of antifungal activity against *Fusarium* spp. during *in vitro* sensitivity tests. The findings of this study provide valuable guidance for establishing the epidemiological cutoff and clinical MIC values for *Fusarium* spp. This study paves the way for future multicenter studies on the treatment of FK with natamycin and chlorhexidine.

## Data Availability

The raw data supporting the conclusions of this article will be made available by the authors, without undue reservation.

## Supplementary material

Supplementary Appendix Figure 1 The geographic distribution of the *Fusarium* species complex. The *F. solani* species complex has the widest distribution, covering 17 provinces (17/20, 85.0%), while the *F. fujikuroi* species complex covers 14 provinces (14/20, 70%), both concentrated in Beijing, Hebei, Inner Mongolia, and Liaoning. They are mainly distributed in Northern China. The pie chart size is directly proportional to the number of strains in the area.

Supplementary Apppendix Table 1 Antifungal susceptibility pattern of *Fusarium* isolates. (Excel-formatted table).

Supplementary Apppendix Table 2 Antifungal susceptibility pattern of Different *Fusarium* spp. Complexes. (Excel-formatted table).

Supplementary Apppendix Table 3 Antifungal susceptibility pattern of *F. solani* and Non-*F. solani.* (Excel-formatted table).
