# Supplementary figures and images for: Antifungal susceptibility and clinical efficacy of chlorhexidine combined with topical ophthalmic medications against Fusarium species isolated from corneal samples
Source: Front Cell Infect Microbiol. 2025 Apr 15;15:1532289. doi: 10.3389/fcimb.2025.1532289 (PMC12037580; doi:10.3389/fcimb.2025.1532289)

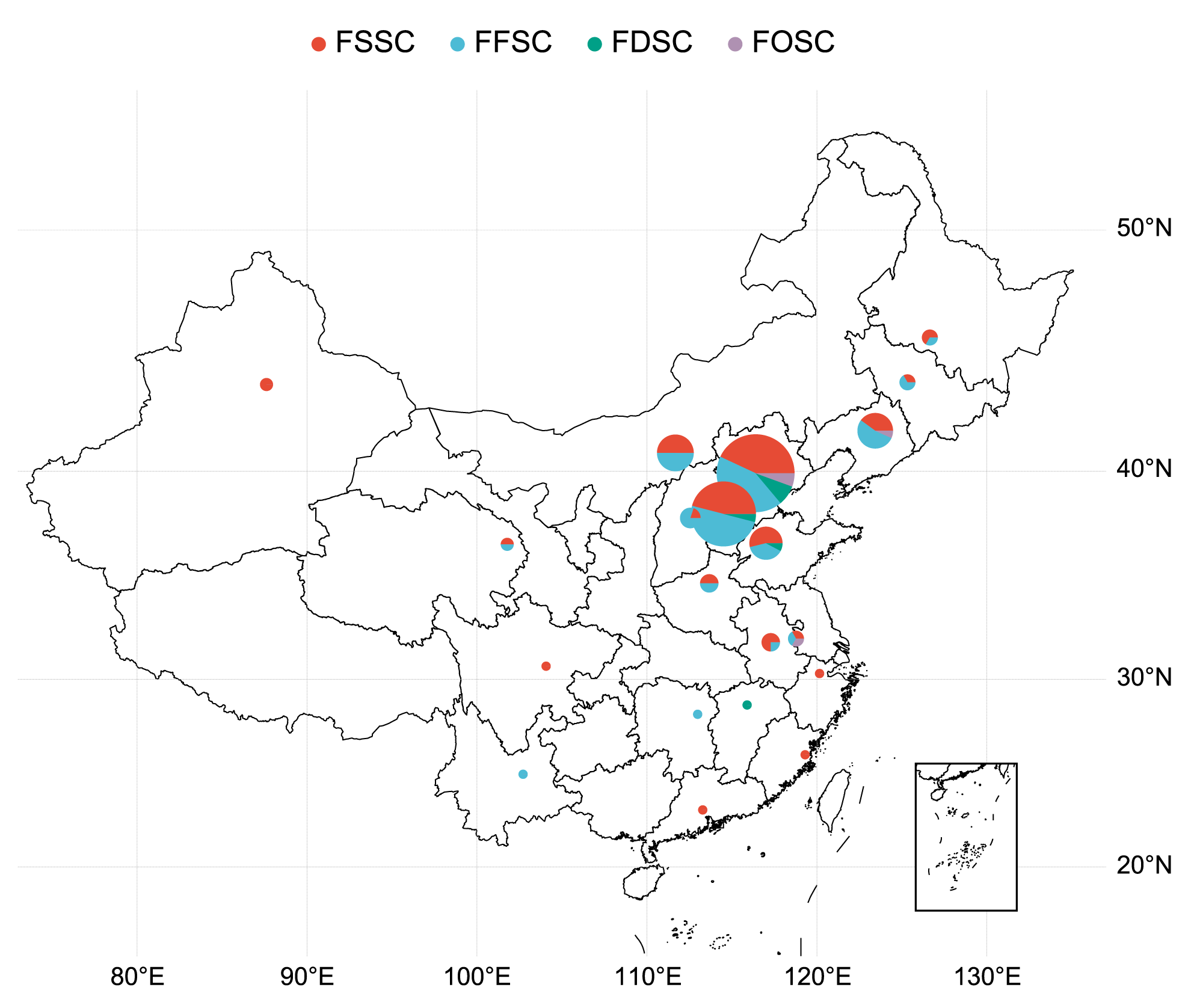

Supplement: Supplementary Appendix Figure 1 — The geographic distribution of the Fusarium species complex. The F. solani species complex has the widest distribution, covering 17 provinces (17/20, 85.0%), while the F. fujikuroi species complex covers 14 provinces (14/20, 70%), both concentrated in Beijing, Hebei, Inner Mongolia, and Liaoning. They are mainly distributed in Northern China. The pie chart size is directly proportional to the number of strains in the area. [file Image1.tif]
